# Experiences and predictors of psychological distress in pregnant women living with HIV

**DOI:** 10.1111/bjhp.12510

**Published:** 2021-02-13

**Authors:** Ifeanyichukwu Anthony Ogueji

**Affiliations:** ^1^ Department of Psychology Faculty of the Social Sciences University of Ibadan Ibadan Oyo State Nigeria

**Keywords:** acceptance of illness, experiences, HIV, meaning in life, pregnant women, psychological distress, self‐compassion

## Abstract

**Objectives:**

To explore the experiences and predictors of psychological distress in pregnant women living with HIV.

**Design:**

A mixed‐methods research design.

**Methods:**

A representative randomly sampled 840 (age range 22–46 years) HIV‐positive pregnant women in Akwa Ibom, Benue, and Rivers States of Nigeria enrolled for the study. Data were collected using standardized questionnaires and in‐depth interviews for 4 months and 3 weeks in 6 HIV treatment centres. Collected data were analysed using IBM SPSS statistics (v. 22.0) and thematic analysis.

**Results:**

The mean score on psychological distress was 17.07 ± 5.86. Multiple regression analysis found a significant joint prediction of meaning in life, self‐compassion, and acceptance of illness on psychological distress, *R* = .64, *R^2^* = .41; *F* (3,828) = 186.18; *p* = .000, with 41% variance explained. Further, there was a significant independent prediction of each predictor at meaning in life (*β* = −.19, *t* = −5.08; *p* = .000), self‐compassion (*β* = −.23, *t* = −5.59; *p* = .000), and acceptance of illness (*β* = −.30, *t* = −7.23; *p* = .000), with acceptance of illness exerting the greatest independent predictive impact. Socio‐demographic variables (age, length of living with HIV, high‐risk state, highest education attained, marital status, and religious affiliation) had no significant contribution to psychological distress. Qualitative analysis found ‘anxious concerns’, ‘depressive reports, loneliness, and regrets’, ‘self‐blame and guilt feelings’, as the experiences of psychological distress, and these experiences were determined by respondents’ socio‐cultural contexts.

**Conclusion:**

These findings emphasize the importance of psychosocial care for HIV‐positive pregnant women.

**Statement of Contribution**

***What is already known about this subject?***

- Pregnant women living with HIV are often psychologically distressed from being pregnant and living with HIV at the same time. Understanding the experiences and predictors of psychological distress can contribute to inform targeted interventions for this population. However, the possible experiences of and factors predicting psychological distress in this population remain scant.
- Using a single research design, related studies attempted to identify the predictors of psychological distress, but they were often inconclusive because of the limited sample sizes that they were conducted on.
- All above is suggestive that sufficient research on the experiences and predictors of psychological distress in a representative sample of pregnant women living with HIV is a meaningful action towards the development of appropriate interventions against psychological distress in the population of interest.

***What does this study add?***

- This mixed‐method study extended related studies by establishing that the study population experiences psychological distress which may be influenced by their wider socio‐cultural context. Therefore, the need for socio‐cultural consideration is imperative in the management/treatment of psychological distress among the study population.
- Another knowledge addition indicated that a model incorporating self‐compassion, acceptance of illness, and meaning in life emerged as a novel model that explains a good and acceptable variance in psychological distress among the population.
- The development of interventions based on this model may support the mitigation of the experiences of psychological distress in this population.

## Background

Among the general population of people living with HIV/AIDS (PLWHA) is a very vulnerable subpopulation who are often psychologically distressed from being pregnant and living with HIV. Psychological distress in HIV‐positive pregnant women is worthy of attention given the fact that related studies have often found high psychological distress to be linked with the extent of caregiving that mothers provide for their infants, and the incidence of mother‐to‐child transmission (MTCT) of HIV (Mbachu, et al., 2020; Qin, Tan, Lu, Cheng, & Nong, 2019; Shouxue et al., 2019). Psychological distress and HIV are often co‐morbid with HIV‐positive pregnant women globally (Chesney & Folkman, 1994; Peltzer, Ogawa, Tusher, Farnan, & Gerkovich, 2016). Psychological distress has also been reported to limit antiretroviral therapy (ART) adherence in this population (Ramlagan et al., 2019), and this is problematic when compared with the normal population of pregnant women or pregnant women with other chronic conditions (Staneva, Morawska, Bogossian, & Wittkowski, 2018).

What is therefore urgently needed is to conduct empirical investigations into the experiences and predictors of psychological distress in pregnant women living with HIV (Bastos, Bellini, Vieira, Campos, & Turato, 2019; Liamputtong, & Haritavorn, 2014; Ngocho et al., 2019). However, very little is known about the experience and predictors of psychological distress in HIV‐positive pregnant women in Nigeria that is currently home to many young females infected with HIV in sub‐Saharan Africa (Kharsany & Karim, 2016). The age range of Nigerian pregnant women most vulnerable to HIV infection has been identified as 36–45 age group, and HIV/AIDS has been identified as among the leading causes of maternal mortality in Nigeria (Khanam, 2019). In 2015, the Nigerian government reported that over 177, 993 pregnant women were living with HIV in Nigeria, and the figures were likely to rise in the coming years (NACA Fact Sheet, 2016). Further, the prevalence of HIV/AIDS among pregnant women in Nigeria has been estimated to be over 26.4%, and this is higher than most other sub‐Saharan African countries (Mbachu et al., 2020; Okonko, Osadebe, Onianwa, & Okereke, 2019). Hence, conducting the current study on the target population in Nigeria is a timely action.

Consequently, this study aimed at exploring the experiences of psychological distress in HIV‐positive pregnant women in Nigeria. The current study also aims to explore some predictors (self‐compassion, meaning in life, and acceptance of illness) of psychological distress in this population.

### Review of literature

#### Self‐compassion and psychological distress

Self‐compassion has often predicted psychological distress and adaptive functioning in vulnerable populations; however, there is not enough information regarding this from a Nigerian perspective of HIV‐positive pregnant women. For instance, Brion, Leary, and Drabkin (2014); found that self‐compassion practices were significantly related to lower stress, anxiety, shame, and better adjustment, among 187 HIV‐positive persons. Dunn, Hanieh, Roberts, and Powrie (2012) demonstrated that elements of self‐compassion significantly reduced anxiety and depression among pregnant women in Australia. Hulsbosch et al. (2020) found that exercises similar to compassionate exercises were contributing factors towards reducing psychological distress among pregnant women.

O’Donovan et al. (2015) found that compassionate acts were imperative in reducing psychological distress among HIV‐positive persons. In South Africa, Kotze (2011) found that attributes of self‐compassion were significantly related to psychological functioning among pregnant women with HIV. Ogueji and Okoloba (2020) demonstrated that being compassionate (kindness to self & others) in thoughts and behaviours fostered adaptive functioning and reduced the risk of suicide among 22 patients with HIV/AIDS. Raque‐Bogdan (2010) showed that compassionate care significantly enhanced hope and positive affect among vulnerable client groups. Ngocho et al. (2019) established that HIV‐shame which is among the concerns of self‐compassion was found to predict the experience of distress in 200 pregnant women living with HIV in Tanzania. Overall, the above studies suggest that self‐compassionate activities may have the potential for supporting normal functioning in HIV‐positive pregnant women. However, these studies leave the question of generalizability effects unanswered.

The above significant impact of self‐compassion on psychological distress is in agreement with the social mentality theory of self‐compassion (Gilbert, 2017; Hermanto & Zuroff, 2016). The theory postulates that mentalities of kindness to self and others, seeking care, and giving care to others characterize self‐compassion (Gilbert, 2005), predicts psychological distress, and other maladjustments in patients (Neff, 2003; Raes, 2010).

#### Meaning in life and psychological distress

Audet, Wagner, and Wallston (2015) determined that meaning in life was significantly correlated with dimensions of distress among persons with HIV in Tennessee. Du et al. (2017) studied 518 PLWHAs and reported that meaning in life fostered adaptive functioning and reduced attributes of psychological distress. Moradmand‐Badie et al. (2014) showed that meaning in life enhanced spirituality and reduced distress in patients with HIV/AIDS. Wang et al. (2016) demonstrated that redefining the meaning of life potentially contributed to reducing psychological distress among women with HIV/AIDS in China. Deko, Asagba, Agberotimi, and Wimberly (2016) reported that meaning in life was significantly imperative for treating the presence of distress in 200 adult patients living with HIV/AIDS. Liamputtong and Haritavorn (2014) reported that HIV‐positive pregnant women found meaning in their unborn child, and this contributed towards reducing psychological distress in them. Although these studies suggest that meaning in life is crucial for managing psychological distress in the study population, the studies, however, stressed the need for replication on a wider sample.

From the above studies, the findings are central to the theoretical argument of the meaning in life theory (Frankl, 1976), which states that the search for meaning is a primary aspect of human existence, and until meaning is found, the likelihood of presenting distress is accelerated (Breitbart et al., 2018).

#### Acceptance of illness and psychological distress

Tartakovsky and Hamama (2011) found that mothers who were accepting of HIV‐positive diagnosis, experienced improved psychological functioning, and declined psychological distress. Mcintosh et al. (2015) established that patients living with HIV that were avoidant of the reality of HIV‐positive diagnosis reported greater psychological distress and HIV disease severity. Zhou et al. (2019) demonstrated that catastrophizing and denial of illness, rather than accepting of illness, were factors contributing to increased symptoms of distress in HIV patients. It was further found that catastrophizing the result of an HIV‐positive diagnosis was based on forming associations between HIV‐positive diagnosis, stigmatization, and death. Penedo et al. (2003) showed that avoidant coping, which is the opposite of acceptance of illness, increased psychological distress in men living with HIV/AIDS. Ristriyani, Rachmawati, and Afiyanti (2018) found that acceptance of illness significantly determined status disclosure, distress, and coping among 235 women living with HIV/AIDS in Indonesia.

The acceptance of self and status after receiving an HIV‐positive diagnosis has been found to boost coping and diminish psychological distress in pregnant women in Uganda (Ashaba et al., 2017). Similarly, Kotze (2011) demonstrated that acceptance of illness fostered wellness and reduced distress in HIV‐positive pregnant women. The acceptance of illness was also found to be informed by the links that patients formed between the HIV status and their daily living. Illness acceptance has been reported to predict preparation for death, reduce distress, and improve adjustment in PLWHAs (Griffin & Rabkin, 1988). The above findings are a strong agreement in the literature that the acceptance of illness could help reduce psychological distress in people living with HIV. However, there is a scarcity of studies to support this from a representative sample of HIV‐positive pregnant women.

Also, the above findings support the relational frame theory (Hayes et al., 2001). The theory submits that forming interactions between events is the basis for accepting events and improving physical and psychological health (Hayes, 2004; Hendriks & Zuroff, 2016; Luciano et al., 2011).

#### Experiences of psychological distress in HIV‐positive pregnant women

Experiences of psychological distress which include confusion about emotions, doubtfulness, ambiguity in functioning, excessive use of defence mechanisms, and fear of vertical transmission of HIV have been commonly identified in pregnant women living with HIV/AIDS (Bastos et al., 2019). In the same study, it was argued that understanding the experiences of psychological distress in this fragile population is underscored for informing appropriate intervention programmes for them.

Peltzer et al. (2016) found that psychological and emotional sufferings, stigmatization, and feelings of loneliness formed the experiences of psychological distress. Kotze (2011) submitted that self‐blame and avoidant coping were among the experiences of psychological distress in HIV‐positive pregnant women. Lastly, Liamputtong and Haritavorn (2014) demonstrated that anxious expression towards reproductive health needs and fear about being unable to breastfeed infants were major experiences of psychological distress in PLWHAs. These studies explored the experiences of psychological distress to mitigate it; however, the studies often employed a single research design, thus limiting its strength in mitigating psychological distress in the study population (Wisdom & Creswell, 2013). Therefore, a mixed‐method study could elaborate on the mitigation of psychological distress in the population.

#### Statement of the problem

Informed by the above literature, the current study argues that psychological distress is a common problem in the general population of pregnant women living with HIV. Therefore, submitting empirical findings on the experiences and predictors of psychological distress is expected to support the treatment/management of psychological distress in the target population.

The predictor variables in the current study were self‐compassion, meaning in life, and acceptance of illness. The predictors were selected because, in related studies where they were each compared against the psychological health of various subgroups of PLWHAs, there were often limited conclusions due to the small sample sizes of these studies (Deko et al., 2016; Ngocho et al., 2019; Ogueji & Okoloba, 2020; Ristriyani et al., 2018). Therefore, studying the predictor variables on psychological distress in a representative sample is significant for providing further data and informing a wider intervention against psychological distress. Thus, the following questions were explored.

### Research questions

Therefore, it was asked:

1. To what extent will self‐compassion, meaning in life, and acceptance of illness have significant joint and independent predictions on psychological distress among HIV‐positive pregnant women?
2. What experiences of psychological distress do HIV‐positive pregnant women have?

Additionally, the study population is one that has subpopulations; consequently, their socio‐demographic characteristics may have implications on how to target interventions to them. Therefore, it was asked:

- To what extent will socio‐demographic variables significantly predict psychological distress among HIV‐positive pregnant women?

### Hypotheses

H_1_ Self‐compassion, meaning in life, and acceptance of illness will have significant joint and independent predictions on psychological distress among HIV‐positive pregnant women.

H_2_ Socio‐demographic variables will have significant joint and independent predictions on psychological distress among HIV‐positive pregnant women.

## Methods

### Design

This was a mixed‐method research design. The quantitative study was a cross‐sectional survey while the qualitative study was an in‐depth interview. The mixed‐method design was informed by the aim of the study which necessitated quantitative and qualitative data to achieve it (Creswell & Hirose, 2019; Wisdom & Creswell, 2013). Both quantitative and qualitative studies were designed to be conducted simultaneously (Creswell & Hirose, 2019). In the quantitative study, the independent variables were self‐compassion, meaning in life, and acceptance of illness, whereas the dependent variable was psychological distress. The qualitative study focused on exploring the experiences of psychological distress encountered by respondents.

### Setting

The study was conducted in six (6) purposively selected HIV treatment centres in Benue, Rivers, and Akwa Ibom States of Nigeria (1 treatment centre in Rivers, 2 in Benue, and 3 treatment centres in Akwa Ibom). Rivers and Akwa Ibom States are among the 36 states in Nigeria, and they are located in the Southern geopolitical zones of Nigeria. Benue State is among the 36 states in Nigeria, and it is located in the Northern geopolitical zone of Nigeria. These states are the three highest in the prevalence of HIV in Nigeria and are currently home to many pregnant women living with HIV in Nigeria (Nigerian HIV/AIDS Indicator & Impact Survey, 2019). The prevalence of HIV is about 5.5, 5.3, and 3.8%, respectively, in Akwa Ibom, Benue, and Rivers States, and this is higher than in other states of Nigeria (UNAIDS Data, 2019).

Three treatment centres were selected in Akwa Ibom State because the state presently accounts for the highest prevalence of HIV in Nigeria. On the other hand, two and one treatment centres were, respectively, selected in Benue and Rivers States because these states have high prevalence as well, but not as high as Akwa Ibom State (UNAIDS Data, 2019). The treatment centres were also selected based on the high number of HIV‐positive pregnant women that were registered there as at when this study was conducted. Another decision that informed the selection of these treatment centres in the states was the preliminary interview conducted with 12 medical personnel that specialized in the treatment of the study population. The interview was designed to elicit information on which HIV treatment centres were most suitable for accessing respondents. The results from the interview showed a greater consensus in the responses of the interviewees that the selected treatment centres in the current study were a suitable setting for accessing a large number of the study population with diverse socio‐demographic backgrounds.

### Sampling & respondents

At the time this study was conducted, a total of 315 pregnant women were registered as receiving ART in the selected HIV treatment centre of Rivers State, 781 in Akwa Ibom State, and 445 in Benue State. The minimum sample size was determined at 10% of the total registered HIV‐positive pregnant women at each treatment centre (Kadam, & Bhalerao, 2010; Ogueji & Okoloba, 2020). A representative sample of 940 willing respondents was randomly sampled in total; however, 894 respondents returned their questionnaires, but 832 respondents with a mean age of 38.86 ± 13.0 (age range 22–46 years) had their questionnaires completed. Therefore, a response rate of 88% was established. Eight respondents (age range 24–44 years) were randomly recruited and interviewed for the qualitative study. The length of living with HIV for the respondents in both studies ranged from 6 months to 24 years. Additionally, of the total respondents in both studies, 313 (37.26%) were married, whereas 527 (62.74%) were either single, separated, or divorced.

Further detail on the demographic information of respondents was presented in Table 1. Participation in this study required that willing respondents were pregnant women medically diagnosed with HIV/AIDS, could communicate using the English language, and registered as a patient receiving antenatal care and antiretroviral therapy (ART) in any of the selected study setting. It was also required that respondents had no major health problems other than HIV to ensure that HIV and pregnancy were the major sources of psychological distress found in respondents. To achieve these, the nurses working with the respondents in each setting reviewed the case file of respondents to assist in the screening of the inclusion criteria.

**Table 1:** Demographic profile of respondents

	*N*	%
Quantitative study		
Religious affiliation		
Christian	529	63.6
Islam	142	17.1
Traditional religion	136	16.3
Other religion/No religion	25	3.0
Highest education attained		
Primary school	166	20.0
Secondary school	545	65.5
Tertiary education	121	14.5
Number of respondent per high‐risk state		
Rivers	185	22.2
Akwa Ibom	418	50.3
Benue	229	27.5
Qualitative study		
Religious affiliation		
Christian	3	37.5
Islam	2	25.0
Traditional religion	2	25.0
Other religion/No religion	1	12.5
Highest education attained		
Primary school	2	25.0
Secondary school	4	50.0
Tertiary education	2	25.0
Number of respondent per high‐risk state		
Rivers	2	25.0
Akwa Ibom	4	50.0
Benue	2	25.0

### Instruments

The qualitative study collected data using in‐depth interviews that had a mean length of 45 min ± 31.3. Based on the literature review (Bastos et al., 2019; Peltzer et al., 2016), the central interview question was ‘In what way does being pregnant and living with HIV cause you distress?’. Where necessary, the interviewer prompted respondents to expand their responses. The interview session was recorded with a mobile phone device. The quantitative study using standardized questionnaires collected data on the socio‐demographic information, that is, age, length of living with HIV, marital status, religious affiliation, highest education attained, and state of recruitment (high‐risk state). It further included the following scales (specifically for this study, respondents were asked to fill out each scale regarding their experiences of being pregnant and living with HIV) which were used to measure the dependent and independent variables, respectively:

The Kessler psychological distress scale (K6), developed by Kessler (2003), was used to measure psychological distress in respondents. The scale consisted of 6 items, and the possible total score ranged from 0 to 24. High scores implied high levels of psychological distress and vice versa. The current study found a reliability coefficient of 0.79 for the psychological distress scale. Next was the HIV meaningfulness scale (HIVMS) developed by Audet et al. (2015). The scale was designed majorly for the general population of PLWHAs. It contained 4 items that asked respondents to rate how meaningful life is despite living with HIV/AIDS. A high level of meaningfulness was interpreted by a high score from respondents and vice versa. A 0.60 reliability coefficient was obtained in the current study for the HIV meaningfulness scale.

The self‐compassion scale (short‐form) authored by Raes, Pommier, Neff, and Van Gucht (2010) was used to measure the self‐compassion of respondents. Twelve (12) items were on the scale, and high scores were translated as high levels of self‐compassion and vice versa. The current study reported a 0.74 reliability coefficient for the self‐compassion scale. Last was the acceptance of illness scale adopted from Nowicki, Krzemkowska, and Rhone (2016). The scale consisted of 8 items that assessed the extent to which respondents were willing to accept and live with the reality of HIV/AIDS. A high score on this scale meant that respondents have accepted to live with HIV and vice versa. A 0.75 reliability coefficient was obtained in the current study for the acceptance of the illness scale.

Before conducting this study, all research instruments were pre‐tested on 55 willing respondents with diverse socio‐demographic backgrounds. These respondents were randomly selected from six HIV treatment centres across four states (Anambra, Lagos, Kaduna, and Kebbi states) in Nigeria that was not designated for the main study. All 55 respondents were pregnant and living with HIV, and they all reported that the research instruments were friendly and easy to use. This, therefore, suggested that the research instruments were suitable for use in the main study.

Another important consideration during the pre‐testing of the instruments was to check for likely correlation among the predictor variables by presenting a zero‐order correlation table from the data collected in the pre‐testing phase. This was informed by scholars who opined that in studies interested in the predictive impacts of a set of variables on other variables, the analysis of zero‐order correlation should be conducted before the predictive testing. These scholars also opined that the zero‐order correlation helps to provide additional information about the predictive validity of the predicting variables (Ark, 2014; Cohen, Cohen, West, & Aiken, 2003; Starks, 2011; Verbeek, 2017).

Acknowledging these scientific suggestions from scholars, the zero‐order correlation results in the pre‐test revealed that the predicting variables were not correlated. Thus, implying that any statistical significance obtained in the predictive testing will not be undermined. A zero‐order correlation analysis was also conducted in the main study and presented in Table 2.

**Table 2:** Zero‐order correlation table showing the relationship among variables

SN	Variables	1	2	3	4	X	SD
1	Meaning in life	–	.28	.21	−.49**	10.47	4.60
2	Self‐compassion		–	.34	−.55**	31.12	12.19
3	Acceptance of illness			–	−.59**	21.16	8.19
4	Psychological distress				–	17.07	5.86

** Correlation is significant at 0.01 level (2‐tailed) *n* = 832.

### Procedure

Ethical approval was obtained from the Ethical Review Board of Akwa Ibom, and Benue States Nigeria Ministry of Health who confirmed that the study followed the Helsinki 1964 ethical declaration. The research purpose was explained to respondents and both written and verbal consents were obtained from respondents before data collection. This study had nine experienced research assistants (postgraduate students of clinical psychology in Nigeria) who were required to complete online ethical training offered by the Centre for Research and Bioethics, Nigeria, before the study commenced.

Willing respondents that met the inclusion criteria were randomly selected in each study setting. Unwilling respondents attributed their unwillingness to mostly time constraints on their parts. Throughout the study, it was ensured that neither risk nor harm was brought to respondents. Confidentiality was also constant throughout the study. Every respondent was free to decline participation in the study without any implication. Data were collected on the clinic days of respondents for four months and three weeks. This was because the respondents could only be accessed on their clinic days in all treatment centres. When respondents received the questionnaire, some filled it out immediately, while some took it to their homes and returned it on their next clinic days. A total of 832 respondents completed the questionnaires in the quantitative study.

For the qualitative study, the in‐depth interviews were conducted in the same modalities in all treatment centres with a total of eight randomly recruited respondents. Specifically, two respondents were interviewed in Rivers State treatment centres, two respondents were interviewed in Benue State treatment centres, and four respondents were interviewed in Akwa Ibom State treatment centres. The qualitative study respondents were not part of the quantitative study to minimize response fatigue (Creswell & Hirose, 2019). All respondents in quantitative and qualitative studies were given incentives, and the research assistants were remunerated after the study. There was a strong restriction for the same respondent to fill out the questionnaire more than once because the record of recruited respondents was closely monitored by the researcher, together with the research assistants and medical personnel (nurses at each treatment centre) who assisted the data collection process at each setting. Also, all respondents were reported to have adequately adhered to instructions guiding their participation in the study. The researcher, research assistants, and nurses were involved in collecting quantitative data because of the large number of respondents whereas the qualitative data were collected by the researcher and supported by female nurses with experiences in conducting qualitative research.

Further, the occurrence of response bias was reduced as all respondents maintained an anonymous identity throughout the study. As part of the ethical considerations, approval was sought and granted to conduct a free group counselling session after the study with all respondents in each setting, given the fact that most respondents were psychologically distressed. The group counselling sessions focused on building resilience and strengthening the social support systems of respondents.

Finally, The IBM SPSS statistics (v.22.0) was used for quantitative data analyses and statistical significance was determined at *p *< .05. Thematic analysis was conducted to identify common themes from the responses supplied by respondents in the qualitative study. As informed by literature (Nowell, Norris, White, & Moules, 2017; Roberts et al., 2019), thematic analysis was most appropriate given the second research question which aimed to explore the experiences of psychological distress. A data‐driven approach was applied to the thematic analysis. In the thematic analysis, recorded interviews were transcribed, after which the transcripts were coded. The transcripts were read and re‐read to identify likely and recurring themes. After this, the next step was aimed to identify and report quotes that were in harmony with identified themes. The thematic analysis was conducted with the qualitative research question kept in mind. After thematic analysis, the qualitative results were presented to two health psychologists, one psychiatrist, and one gynaecologist who were purposively selected due to their broad experiences in treating the study population at a health facility in Nigeria. The experts validated the qualitative results based on their expertise. Two respondents in the qualitative study were also given feedback from the qualitative analysis as a part of the validity check. The quantitative data were analysed by a postgraduate student in psychometrics whereas the qualitative data were analysed by the researcher, female nurses, and a postgraduate student in clinical psychology. All analysts were experienced in conducting quantitative and qualitative data analyses (however, they were cautious enough to prevent their expertise from influencing the results).

## Results

### Quantitative results

Table 2 showed that meaning in life (*r* = −.49; *df* = 830; *p *< .01), self‐compassion (*r* = −.55; *df* = 830; *p *< .01), and acceptance of illness (*r* = −.59; *df* = 830; *p *< .01), had significant negative relationships with psychological distress in this study. A negative relationship suggests that respondents that scored higher on these variables (meaning in life, self‐compassion, and acceptance of illness) tend to experience lower levels of psychological distress. The table also showed that the independent variables were not correlated with one another.

### Testing of hypotheses

#### Hypothesis 1

It was hypothesized that self‐compassion, meaning in life, and acceptance of illness will have significant joint, and independent predictions on psychological distress among HIV‐positive pregnant women. This was tested using multiple regression analysis.

Table 3 showed that meaning in life, self‐compassion, and acceptance of illness jointly predicted psychological distress, *R* = .64, *R* ^2^ = .41; *F*(3,828) = 186.18; *p* = .000. Further, it was observed that the predictor variables jointly accounted for 41% variance in psychological distress (*R* = .64; *R* ^2^ = .41), whereas other variables not considered in this study accounted for the remaining 59% variance. This implied that 41% of the variation in psychological distress among respondents can be explained by the model (meaning in life, self‐compassion, and acceptance of illness), and this is considered to be a good model. It is further implied that 59% of the variation in psychological distress was unexplained; therefore, the addition of other independent variables may be relevant for enhancing the model fit.

**Table 3:** Summary of multiple regression table showing the independent and joint predictive strength of meaning in life, self‐compassion, and acceptance of illness on psychological distress among respondents

Predictors	*Β*	*T*	*p*	*R*	*R* ^2^	*F*	*p*
Meaning in life	−.19	−5.08	.000				
Self‐compassion	−.23	−5.59	.000				
				.64	.41	186.18	.000
Acceptance of Illness	−.30	−7.23	.000				

Criterion variable = Psychological Distress.

The independent contribution of each predictor variable showed that meaning in life (*β* = −.19, *t* = −5.08; *p* = .000), self‐compassion (*β* = −.23, *t* = −5.59; *p* = .000), and acceptance of illness (*β* = −.30, *t* = −7.23; *p* = .000) were found to show significant independent contributions on psychological distress, with acceptance of illness showing the greatest independent contribution, followed by self‐compassion, and after which was followed by meaning in life. It is further seen that each predictor variable had a significant negative weight; this is, therefore, an indication that respondents that scored higher on these predictors reported lower psychological distress. The stated hypothesis that self‐compassion, meaning in life, and acceptance of illness will have significant joint, and independent predictions on psychological distress among HIV‐positive pregnant women were thus accepted in this study.

#### Hypothesis 2

It was hypothesized that socio‐demographic variables will have significant joint and independent predictions on psychological distress among HIV‐positive pregnant women.

Table 4 showed that age, length of living with HIV, high‐risk state, highest education attained, marital status, and religious affiliation had no significant joint prediction on psychological distress, *R* = .09, *R* ^2^ = .01; *F*(6,825) = 1.24; *p* = .29. Further, it was observed that the predictor variables jointly accounted for 1% variance in psychological distress (*R* = .09; *R* ^2^ = .01), whereas other variables not considered in this study accounted for the remaining 99% variance.

**Table 4:** Summary of multiple regression table showing the independent and joint predictions of socio‐demographic variables on psychological distress among respondents

Predictors	*Β*	*T*	*p*	*R*	*R* ^2^	*F*	*p*
Age	–.05	–1.29	.20				
Length of living with HIV	–.07	–1.85	.07				
				.09	.01	1.24	.29
High‐risk state	–.04	–1.02	.31				
Highest education attained	.02	.62	.54				
Marital status	.03	.90	.37				
Religious affiliation	‐.01	–.34	.73				

Criterion variable = Psychological Distress.

The independent contribution of each predictor variable showed that age (*β* = −.05, *t* = −1.29; *p* = .20), length of living with HIV (*β* = −.07, *t* = −1.85; *p* = .07), high‐risk state (*β* = −.04, *t* = −1.02; *p* = .31), highest education attained (*β* = .02, *t* = .62; *p* = .54), marital status (*β* = .03, *t* = .90; *p* = .37), and religious affiliation (*β* = −.01, *t* = −.34; *p* = .73) had no significant independent contribution to psychological distress. The stated hypothesis that socio‐demographic variables will have significant joint and independent predictions on psychological distress among HIV‐positive pregnant women was thus rejected in this study.

### Qualitative results

This section presented the experiences of psychological distress among 8 respondents as revealed by thematic analysis. Responses were labelled with the state that the respondent was selected from and the age of the respondent. The following themes were created:

#### Anxious concerns

Anxious concern was the first theme created, as 6 respondents reported that being pregnant and living with HIV often triggered anxious concerns, fears, and excess worries about their unborn child, sexual health, and placement on lifetime medications. These are evident in the following responses:

This is something I never wished myself in life because I have just one sexual partner. But since I took in, and found out that my partner had infected me with HIV, I get worried and scared daily about the chances of me not infecting my unborn baby. I need God’s grace at this time [shivers]. (Benue state, 31 years of age)

I am too young to be living with HIV. I have not enjoyed much of my sexual life…I got diagnosed with HIV about 7 months ago, and I do not care much about my current pregnancy. One of the greatest concerns for me now is that I am honestly anxious that living with HIV may affect my enjoyment of my sexual life now, and also in the future if I don’t die from this infection. (Akwa Ibom state 24 years of age)

Another one said:

For me, I don’t even know where to start from as this is my second pregnancy…I have lost interests in most of the things I love doing, and withdrawn from social interactions…Most times, when I am alone in my room, I begin to think with fear about the fact that I now live on medications for the rest of my life. This sometimes even costs me my sleep at night. (Akwa Ibom state, 28 years of age)

#### Depressive reports, loneliness, and regrets

A respondent stated that her major experience was depressive experiences and loneliness because she felt that she was alone in this world. This was observed in the following response:

It is like nobody cares, I feel like I am alone in this world now, and this is because living with HIV, and being pregnant is the saddest thing I have ever known….I even tried to abort my unborn baby one day, but my boyfriend stopped me…My boyfriend keeps assuring me that he will always be there for me because he infected me. But I still feel like he will leave me with this mess…I am not just happy at all, and I do not have anyone to support me. (Rivers state, 32 years of age)

Another respondent who expressed depressive symptoms also showed that she had regrets, because of the impact of being pregnant and living with HIV. However, she highlighted in her response that she drew support from church programmes and her boss at work. This may illustrate the importance of spirituality and social support for the study population.

What is the use of life when in my kind of pregnant condition together with having an HIV‐positive status?…I no dey happy with everything [I am not happy with everything]…I would have not been HIV‐positive today if only I listened to my mother…but I just thank God for my church where I feel better each time I attend church programs. I also thank God for my boss at work for being supportive. (Benue state, 36 years of age)

Contrarily, a respondent indicated that she had depressive symptoms from the onset of living with HIV; however, knowing that she will soon be a mother was enough happiness for her. Theoretically (based on the meaning in life theory), this may imply that the respondent found meaning in motherhood/her unborn child and this probably contributed to giving her happiness.

When I got the news that I was positive, it was hard for me ooo my brother! [Emphasizes]…I lost food appetite for more than a week, and even attempted to kill myself. All these were last year, and parts of early this year…But currently, I am happy to know that I will soon be a mother, and giving birth to my unborn child HIV‐free is the only thing I hope for right now [Smiles]. (Akwa Ibom state, 34 years of age)

#### Self‐blame and guilt feelings

This was the last theme created showing that some respondents submitted self‐blame and guilt feelings as their major experiences of psychological distress. Theoretically, this theme endorsed the importance of self‐compassion in reducing psychological distress, given that the social mentality theory argues that being compassionate to the self and others can prevent self‐blame and guilt feelings. Illustrative response supporting this theme was:

Whenever I am alone, I keep blaming myself for being the cause of what I am going through today because I be runz babe [because I am a commercial sex worker]….Although I do my best to take my medications and attend my clinic appointments, it has still not been easy due to money and stigma…Because of these, I always tell myself that if I give birth to my child positive, it is my fault. (Akwa Ibom state, 30 years of age)

Another respondent endorsed this theme. Additionally, she highlighted the role of her socio‐cultural context (that is, being from a family where Islam was strongly practiced made it hard for her to disclose her HIV status). This, therefore, is indicative that the socio‐cultural context of the study population may contribute to their experiences of psychological distress.

Being from a strong Islamic family, it was very hard for me to disclose my status, and it became worse for me after I found out that I was pregnant after having premarital sex [Blinks Eyes]…As a result, I am not very comfortable to seek help with my current state, and most times I feel guilty that I am not taking the right actions for me or my unborn child. (Rivers state, 44 years of age)

## Discussion and implications for clinical practice

The quantitative insights from this study showed that meaning in life, self‐compassion, and acceptance of illness had significant negative associations with psychological distress as determined by Pearson *r*. Further, multiple regression analysis revealed that meaning in life, self‐compassion, and acceptance of illness exerted significant independent predictions on psychological distress. The same analysis identified that the predictors had a significant joint prediction, with 41% variance explained in psychological distress. Given this, psychologists have argued that due to the complexity of human behaviours, a model that accounts for a minimum of 5% variance is acceptable, while some similar scholars argued that models that account for 10% variance or greater are considered an acceptable model (Itaoka, 2012; Moksony, 1999; Pagano, 1994). These arguments from behavioural science scholars thus suggest that the model in the current study is acceptable.

Additionally, this study explored the socio‐demographic predictors of psychological distress because this may have a practical implication on how to target health programmes to the subpopulations of the study population. A multiple regression analysis found that age, length of living with HIV, high‐risk state, highest education attained, marital status, and religious affiliation had no significant joint nor independent prediction on psychological distress. One per cent variance in psychological distress was explained by the socio‐demographic model. Consequently, an implication of this could be that the socio‐demographic variables in this study may not be imperative for consideration when designing interventions for the study population.

Although the current study was not an intervention one, the findings from the quantitative study demonstrate a promising benefit that the study population may report from a holistic intervention that incorporates the significant predictors together. Such a promising benefit may be that a single intervention programme that incorporates the predictors together could potentially mitigate psychological distress and promote healthy functioning in the study population. Quantitative findings thus aligned with related studies that have demonstrated that these predictors are imperative for reducing psychological distress (Brion et al., 2014; Deko et al., 2016; Hulsbosch et al., 2020; Ristriyani et al., 2018). Further, the quantitative findings are a theoretical consensus with the social mentality theory of self‐compassion, meaning in life theory, and relational frame theory that guided this study (Frankl, 1976; Gilbert, 2017; Hayes et al., 2001; Hermanto & Zuroff, 2016).

A possible explanation for the significant findings in the quantitative study could be that the predictors are relevant variables whose absences can trigger psychological distress in vulnerable populations. An additional novelty of the current study is that the study was among the first to demonstrate the important predictive impact of a single model comprising self‐compassion, meaning in life, and acceptance of illness; on psychological distress in PLWHAs. This is supportive of the absence of this single model in the literature (Deko et al., 2016; Hulsbosch et al., 2020; Ogueji & Okoloba, 2020).

The qualitative study which explored the experiences of psychological distress reported by respondents found the themes; anxious concerns, depressive reports, loneliness, and regrets, self‐blame, and guilt feelings. Respondents who reported anxious concerns often submitted that their major anxieties were about their sexual health, unborn child, and lifetime placement on medications. A respondent with anxious concern, for instance, mentioned that she was anxious about the fact that she is now on lifetime placement on medications, and consequently, she had lost interest in the activities she loves and had withdrawn from social interactions.

In the second theme, a respondent who although was depressed reported that knowing that she would soon be a mother was enough happiness for her. This suggests that pregnancy could be a source of meaning or hope for HIV‐positive pregnant women. Another respondent who experienced depression and regrets mentioned that she felt relieved each time she attended religious programmes at her church. It may thus imply that spirituality may help buffer against psychological distress in the study population. In the third theme, a respondent mentioned that she experienced stigmatization which prevented her from adhering to healthy behaviours while pregnant, and this contributed to her reports of self‐blame. This report reflects that the experience of stigmatization breeds psychological distress, and this is a barrier to healthy behaviour practice in the study population. Another respondent in the third theme mentioned that her initial source of distress was religiously imposed, that is, being from an Islamic family made it uneasy for her to disclose the HIV status and seek help, consequently, she felt that she was not taking appropriate actions for herself and the unborn child. This, therefore, suggests that the socio‐cultural context of the study population may play a very critical role in the psychological distress reported by them, and this context may need to be considered when developing interventions for them.

These qualitative insights indicate that pregnant women living with HIV may have experiences of psychological distress which takes a toll on their physical, mental, and social functioning. Therefore, this was in agreement with related studies that reported an implication similar to that in the current study (Bastos et al., 2019; Kotze, 2011; Liamputtong, & Haritavorn, 2014; Peltzer et al., 2016). A probable reason for the reported experiences of psychological distress among respondents in this study could be attributed to African socio‐cultural factors associated with HIV such as that HIV is a disease that afflicts immoral people (Muoghalu & Jegede, 2013). Therefore, the intention to avoid such negative societal perceptions about HIV could have contributed to the experience of psychological distress reported by respondents.

Lastly, this mixed‐method design has enabled an understanding of the experiences of psychological distress from the perspective of the study population. It has also revealed some significant predictors of psychological distress among the study population. Therefore, the mixed‐method design implies that the experiences of psychological distress place limitations on the normal functioning of pregnant women living with HIV, and the significant predictors in this study may help mitigate the experiences of psychological distress among them. Additionally, the findings from this mixed‐method design are argued to have endorsed the data reported in other parts of the world given the consensus in findings with studies from the Netherlands (Hulsbosch et al., 2020), Indonesia (Ristriyani et al., 2018), Brazil (Bastos et al., 2019), and the United Kingdom (O’Donovan et al., 2015).

### Strengths and limitations of the study

This study was a mixed‐method design and drew a large sample size from the major high‐risk states of HIV infection in Nigeria. These are strengths over related studies that often adopted a single research design or studied a relatively small sample size. Further, the large sample size in the current study strengthens the generalizability of findings. This study required that respondents responded using the English language because the researcher was unable to access translation services. However, some respondents in the qualitative study responded using the Pidgin English language in the middle of the interview. This, therefore, suggest that respondents responded in the interview from their perspectives, and this is among the importance of qualitative research (wisdom & Creswell, 2013). On the other hand, the current study could have encountered minimal response biases during the data collection.

### Recommendation and conclusion

Conclusively, this study established that there are a significant joint and independent predictive impact of meaning in life, self‐compassion, and acceptance of illness on psychological distress among pregnant women living with HIV. The study also concluded that the experiences of psychological distress are a common health concern that co‐morbid with HIV in HIV‐positive pregnant women.

The current study recommends the need for psycho‐education programmes that aim to reduce public stigmatization of pregnant women living with HIV. There is also the need to develop an intervention that is tailored to the reports of anxious concerns, withdrawal from social interactions, depressive reports, loneliness, self‐blame, regret, and guilt feelings that were found in the qualitative insights of this study. Such intervention should be a holistic one incorporating a clear life meaning, self‐compassion, and acceptance of illness. These recommendations, therefore, suggest the relevance of a multidisciplinary team that includes Clinical Health Psychologists, for the treatment/management of HIV‐positive pregnant women.

Finally, there is a need for further studies that support the mitigation of psychological distress in the study population by considering the potential roles of the psychosocial contexts of the study population.

## Conflict of interest

The author has no conflict of interest to declare.

## Funding

Funding received was in the form of an open access publication funding by the British Psychological Society (BPS).

## Author contributions

Ifeanyichukwu Anthony Ogueji (Conceptualization; Data curation; Formal analysis; Investigation; Methodology; Supervision; Validation; Writing – original draft; Writing – review & editing).

## Data Availability

The data associated with this study are available from the corresponding author upon request.

## References

[bjhp12510-bib-0001] Ark, N. (2014). Zero‐order relationships. In A. C. Michalos (Ed.), Encyclopedia of quality of life and wellbeing research. Dordrecht, Netherland: Springer. 10.1007/978-94-007-0753-5_3306

[bjhp12510-bib-0002] Ashaba, S., Kaida, A., Coleman, J. N., Burns, B. F., Dunkley, E., O’Neil, K., … Matthews, L. T. (2017). Psychosocial challenges facing women living with HIV during the perinatal period in rural Uganda. PLoS One, 12, e0176256. 10.1371/journal.pone.0176256 28459866 PMC5411062

[bjhp12510-bib-0003] Audet, C. M., Wagner, L. J., & Wallston, K. A. (2015). Finding meaning in life while living with HIV: Validation of a novel HIV meaningfulness scale among HIV‐infected participants living in Tennessee. BMC Psychology, 3(1), 15. 10.1186/s40359-015-0070-7 25945254 PMC4419455

[bjhp12510-bib-0004] Bastos, R. A., Bellini, N. R., Vieira, C. M., Campos, C. J. G., & Turato, E. R. (2019). Psychological phases of pregnant women with HIV: A qualitative study in a hospital. Revista Bioética, 27(2), 281–288. 10.1590/1983-80422019272311

[bjhp12510-bib-0005] Breitbart, W., Pessin, H., Rosenfeld, B., Applebaum, A. J., Lichtenthal, W. G., Li, Y., … Fenn, N. (2018). Individual meaning‐centered psychotherapy for the treatment of psychological and existential distress: A randomized controlled trial in patients with advanced cancer. Cancer, 124, 3231–3239. 10.1002/cncr.31539 29757459 PMC6097940

[bjhp12510-bib-0006] Brion, J. M., Leary, M. R., & Drabkin, A. S. (2014). Self‐compassion and reactions to serious illness: The case of HIV. Journal of Health Psychology, 19(2), 218–229. 10.1177/1359105312467391 23300046 PMC4355940

[bjhp12510-bib-0007] Chesney, M. A., & Folkman, S. (1994). Psychological impact of HIV disease and implications for intervention. Psychiatric Clinics, 17(1), 163–182. 10.1016/S0193-953X(18)30136-9 8190663

[bjhp12510-bib-0008] Cohen, J., Cohen, P., West, S. G., & Aiken, L. S. (2003). Applied multiple regression/correlation analysis for the behavioral sciences (3rd ed.). Mahwah, NJ: Erlbaum.

[bjhp12510-bib-0009] Creswell, J. W., & Hirose, M. (2019). Mixed methods and survey research in family medicine and community health. . Family Medicine and Community Health, 7(2), e000086. 10.1136/fmch-2018-000086 32148709 PMC6910743

[bjhp12510-bib-0010] Deko, A. O., Asagba, R. B., Agberotimi, S. F., & Wimberly, C. (2016). Personal features and wellbeing as predictors of meaning in life among people living with HIV/AIDS (PLWHAs) in Nigeria. Edorium Journal of Psychology, 2, 1–7. 10.5348/P13-2016-7-OA-1

[bjhp12510-bib-0011] Du, H., Li, X., Chi, P., Zhao, J., & Zhao, G. (2017). Meaning in life, resilience, and psychological well‐being among children affected by parental HIV. AIDS Care, 29, 1410–1416. 10.1080/09540121.2017.1307923 28343403

[bjhp12510-bib-0012] Dunn, C., Hanieh, E., Roberts, R., & Powrie, R. (2012). Mindful pregnancy and childbirth: Effects of a mindfulness‐based intervention on women’s psychological distress and well‐being in the perinatal period. Archives of Women's Mental Health, 15(2), 139–143. 10.1007/s00737-012-0264-4 22382281

[bjhp12510-bib-0013] Frankl, V. (1976). The unconscious god. New York, NY: Touchstone.

[bjhp12510-bib-0014] Gilbert, P. (2005). Compassion and cruelty: A biopsychosocial approach. In P. Gilbert (Ed.), Compassion: Conceptualizations, research and use in psychotherapy (pp. 9–74). London, UK: Brunner‐Routledge.

[bjhp12510-bib-0015] Gilbert, P. (2017). Compassionate as a social mentality: An evolutionary approach. In P. Gilbert (Ed.), Compassion: Concepts, research and applications (pp. 31–68). London, UK: Routledge/Taylor & Francis Group.

[bjhp12510-bib-0016] Griffin, K. W., & Rabkin, J. G. (1988). Perceived control over illness, realistic acceptance, and psychological adjustment in people with AIDS. Journal of Social and Clinical Psychology, 17(4), 407–424. 10.1521/jscp.1998.17.4.407

[bjhp12510-bib-0017] Hayes, S. C. (2004). Acceptance and commitment therapy, relational frame theory, and the third wave of behavioral and cognitive therapies. Behavior Therapy, 35, 639–665. 10.1016/S0005-7894(04)80013-3 27993338

[bjhp12510-bib-0018] Hayes, S. C., Barnes‐Holmes, D., & Roche, B. (2001). Relational frame theory: A post‐Skinnerian account of human language and cognition. New York, NY: Kluwer Academic/Plenum Publishers. 10.1016/s0065-2407(02)80063-5 11605362

[bjhp12510-bib-0019] Hendriks, A. L., Barnes‐Holmes, Y., McEnteggart, C., De Mey, H. R., Janssen, G. T., & Egger, J. I. (2016). Understanding and remediating social‐cognitive dysfunctions in patients with serious mental illness using relational frame theory. Frontiers in Psychology, 7, 143. 10.3389/fpsyg.2016.00143 26903935 PMC4751275

[bjhp12510-bib-0020] Hermanto, N., & Zuroff, D. C. (2016). The social mentality theory of self‐compassion and self‐reassurance: The interactive effect of care‐seeking and caregiving. The Journal of Social Psychology, 156(5), 523–535. 10.1080/00224545.2015.1135779 26736073

[bjhp12510-bib-0021] Hulsbosch, L. P., Nyklíček, I., Potharst, E. S., Meems, M., Boekhorst, M. G., & Pop, V. J. (2020). Online mindfulness‐based intervention for women with pregnancy distress: Design of a randomized controlled trial. BMC Pregnancy and Childbirth, 20(1), 1–10. 10.1186/s12884-020-2843-0 PMC7069182 32169030

[bjhp12510-bib-0022] Itaoka, K. (2012, April). Regression and interpretation low R‐Squares. In Proceedings of the presentation at Social Research Network 3rd Meeting, Noosa. Mizuho Information and Research Institute Inc.

[bjhp12510-bib-0023] Kadam, P., & Bhalerao, S. (2010). Sample size calculation. International Journal of Ayurveda Research, 1(1), 55. 10.4103/0974-7788.59946 20532100 PMC2876926

[bjhp12510-bib-0024] Kessler, R. C., Barker, P. R., Colpe, L. J., Epstein, J. F., Gfroerer, J. C., Hiripi, E., … Zaslavsky, A. M. (2003). Screening for serious mental illness in the general population. Archives of General Psychiatry, 60(2), 184–189. 10.1001/archpsyc.60.2.184 12578436

[bjhp12510-bib-0025] Khanam, S. (2019). Prevalence of HIV infection among pregnant women attending Ajiko medical clinic, Damaturu, Nigeria. Journal of Clinical Research in HIV AIDS and Prevention, 3, 7. 10.14302/issn.2324-7339.jcrhap-19-2746

[bjhp12510-bib-0026] Kharsany, A. B., & Karim, Q. A. (2016). HIV infection and AIDS in sub‐Saharan Africa: Current status, challenges and opportunities. The Open AIDS Journal, 10, 34. 10.2174/1874613601610010034 27347270 PMC4893541

[bjhp12510-bib-0027] Kotze, M. (2011). The coping strategy used over a two‐year period by HIV‐positive women who were diagnosed during pregnancy. A dissertation submitted to Department of Psychology, as partial fulfilment for the requirements of a Masters of Arts degree in Research Psychology. http://hdl.handle.net/2263/29980

[bjhp12510-bib-0028] Liamputtong, P., & Haritavorn, N. (2014). My life as Mae Tid Chua [mothers who contracted HIV disease]: Motherhood and women living with HIV/AIDS in central Thailand. Midwifery, 30(12), 1166–1172. 10.1016/j.midw.2014.04.003 24812033

[bjhp12510-bib-0029] Luciano, C., Ruiz, F. J., Torres, R. M. V., Martín, V. S., Martínez, O. G., & López, J. C. L. (2011). A relational frame analysis of defusion interactions in acceptance and commitment therapy. A preliminary and quasi‐experimental study with at‐risk adolescents. International Journal of Psychology and Psychological Therapy, 11(2), 165–182.

[bjhp12510-bib-0030] Mbachu, I. I., Ejikunle, S. D., Anolue, F., Mbachu, C. N., Dike, E., Ejikem, E., & Okeudo, C. (2020). Relationship between placenta malaria and mother to child transmission of HIV infection in pregnant women in South East Nigeria. Malaria Journal, 19(1), 1–8. 10.1186/s12936-020-03171-2 32103782 PMC7045610

[bjhp12510-bib-0031] McIntosh, R. C., Hurwitz, B. E., Antoni, M., Gonzalez, A., Seay, J., & Schneiderman, N. (2015). The ABCs of trait anger, psychological distress, and disease severity in HIV. Annals of Behavioral Medicine, 49(3), 420–433. 10.1007/s12160-014-9667-y 25385204 PMC4623323

[bjhp12510-bib-0032] Moksony, F. (1999). Small is beautiful. The use and interpretation of R2 in social research. Szociológiai Szemle, Special issue, 130‐138.

[bjhp12510-bib-0033] Moradmand‐Badie, B., Moayedi‐Nia, S., Foroughi, M., Dejman, M., Nikzad, R., Akhlaghkhah, M., … Mwanri, L. (2014). HIV positive patients’ experiences on receiving an HIV positive test: An Iranian‐qualitative study. American Journal of Epidemiology and Infectious Disease, 2(1), 47–55. 10.12691/ajeid-2-1-9

[bjhp12510-bib-0034] Muoghalu, C. O., & Jegede, S. A. (2013). Perception of HIV/AIDS among the Igbo of Anambra State, Nigeria. SAHARA‐J: Journal of Social Aspects of HIV/AIDS, 10(1), 42–54. 10.1080/17290376.2013.807052 PMC3914455 23808412

[bjhp12510-bib-0035] NACA Fact Sheet . (2016). Fact sheet: HIV prevention program – NACA Nigeria. Retrieved from https://naca‐gov.ng/fact‐sheet‐hiv‐prevention‐program/

[bjhp12510-bib-0036] Neff, K. D. (2003). Development and validation of a scale to measure self‐compassion. Self and Identity, 2, 223–250. 10.1080/15298860309027

[bjhp12510-bib-0037] Ngocho, J. S., Watt, M. H., Minja, L., Knettel, B. A., Mmbaga, B. T., Williams, P. P., & Sorsdahl, K. (2019). Depression and anxiety among pregnant women living with HIV in Kilimanjaro region, Tanzania. PLoS One, 14(10), e0224515. 10.1371/journal.pone.0224515 31671160 PMC6822761

[bjhp12510-bib-0038] Nigerian HIV/AIDS Indicator & Impact Survey . (2019). National Summary Sheet. Retrieved from https://www.naiis.ng/resource/factsheet/NAIIS%20PA%20NATIONAL%20FACTSHEET%20FINAL.pdf

[bjhp12510-bib-0039] Nowell, L. S., Norris, J. M., White, D. E., & Moules, N. J. (2017). Thematic analysis: Striving to meet the trustworthiness criteria. International Journal of Qualitative. Methods, 16(1), 160940691773384. 10.1177/1609406917733847

[bjhp12510-bib-0040] Nowicki, A., Krzemkowska, E., & Rhone, P. (2016). Acceptance of illness after surgery in patients with breast cancer in the early postoperative period. Polish Journal of Surgery, 87(11), 539–550. https://10.1515/pjs‐2016‐0001 10.1515/pjs-2016-0001 26816401

[bjhp12510-bib-0041] O'Donovan, A., Gibson, S., & Gill, E. (2015). Compassion‐focused therapy for people living with HIV: Pilot of a mindfulness and compassion‐based cognitive therapy group. HIV Medicine, 16, 63.

[bjhp12510-bib-0042] Ogueji, A. I., & Okoloba, M. M. (2020). Compassion‐focused therapy (CFT) as an intervention against suicidal ideation in newly diagnosed people living with HIV/AIDS (PLWHA) attending a Nigerian maternity teaching hospital. Global Psychiatry, 3(1), 104–112. 10.2478/gp-2020-0012

[bjhp12510-bib-0043] Okonko, I. O., Osadebe, A. U., Onianwa, O., & Okereke, S. (2019). Prevalence of HIV in a cohort of pregnant women attending a tertiary hospital in Ibadan, Nigeria. Immunology and Infectious Diseases, 7(1), 7–12. https://10.13189/iid.2019.070102

[bjhp12510-bib-0044] Pagano, P. R. (1994). Understanding statistics in the behavioral sciences. (4th, ed., p. 496). Minneapolis, MN: West Publishers.

[bjhp12510-bib-0045] Peltzer, J. N., Ogawa, L., Tusher, S., Farnan, R., & Gerkovich, M. M. (2016). A qualitative description of HIV‐infected African American women’s experiences of psychological distress, and their coping strategies. Journal of the Association of Nurses in AIDS Care, 28(2), 226–237. 10.1016/j.jana.2016.09.010 27771179

[bjhp12510-bib-0046] Penedo, F. J., Gonzalez, J. S., Davis, C., Dahn, J., Antoni, M. H., Ironson, G., … Schneiderman, N. (2003). Coping and psychological distress among symptomatic HIV+ men who have sex with men. Annals of Behavioral Medicine, 25(3), 203. 10.1207/S15324796ABM2503_06 12763715

[bjhp12510-bib-0048] Qin, S., Tan, Y., Lu, B., Cheng, Y., & Nong, Y. (2019). Survey and analysis for impact factors of psychological distress in HIV‐infected pregnant women who continue pregnancy. The Journal of Maternal‐Fetal & Neonatal Medicine, 32(19), 3160–3167. 10.1080/14767058.2018.1459550 29764247

[bjhp12510-bib-0049] Raes, F. (2010). Rumination and worry as mediators of the relationship between self‐compassion and depression and anxiety. Personality and Individual Differences, 48, 757–761. 10.1016/j.paid.2010.01.023

[bjhp12510-bib-0050] Raes, F., Pommier, E., Neff, K. D., & Van Gucht, D. (2010). Construction and factorial validation of a short form of the self‐compassion scale. Clinical Psychology and Psychotherapy, 18, 250–255. 10.1002/cpp.702 21584907

[bjhp12510-bib-0051] Ramlagan, S., Rodriguez, V. J., Peltzer, K., Ruiter, R. A., Jones, D. L., & Sifunda, S. (2019). Self‐reported long‐term antiretroviral adherence: A longitudinal study among HIV infected pregnant women in Mpumalanga. South Africa. AIDS and Behavior, 23(9), 2576–2587. 10.1007/s10461-019-02563-z 31228026 PMC6766468

[bjhp12510-bib-0052] Raque‐Bogdan, T. L. (2010). Self‐compassion, hope, and well‐being of women experiencing primary and secondary infertility: an application of the biopsychosocial model (Doctoral Dissertation). Retrieved from http://hdl.handle.net/1903/11075

[bjhp12510-bib-0053] Ristriyani, R., Rachmawati, I. N., & Afiyanti, Y. (2018). Status disclosure and the acceptance of women living with HIV. Enfermeria Clinica, 28, 195–198. 10.1016/S1130-8621(18)30066-4 29650185

[bjhp12510-bib-0054] Roberts, K., Dowell, A., & Nie, J. B. (2019). Attempting rigour and replicability in thematic analysis of qualitative research data; a case study of codebook development. BMC Medical Research Methodology, 19(1), 66. 10.1186/s12874-019-0707-y 30922220 PMC6437927

[bjhp12510-bib-0055] Shouxue, Q. X., Tan, J. K., Shi, R. G., Wu, H., Nong, Y., Cheng, Y., & Lu, B. (2019). Interventions for prevention of mother‐to‐child HIV transmission in regions with a high HIV/AIDS prevalence in Guangxi Zhuang autonomous region: Success rate, timeliness, and patient compliance. Journal of Third Military Medical University, 40, 1704–1710.

[bjhp12510-bib-0056] Staneva, A. A., Morawska, A., Bogossian, F., & Wittkowski, A. (2018). Maternal psychological distress during pregnancy does not increase the risk for adverse birth outcomes. Women and Health, 58(1), 92–111. 10.1080/03630242.2017.1282395 28095254

[bjhp12510-bib-0057] Starks, P. B. (2011). Chapter 7 correlation and association. In SticiGuti: Retrieved from http://www.stat.berkeley.edu/‐stark/sticigui/text/correlation.htm

[bjhp12510-bib-0058] Tartakovsky, E., & Hamama, L. (2011). Mothers’ acceptance‐rejection of their children infected with HIV: The role of the mothers’ social axioms, psychological distress, and relationships with the partner. Journal of Pediatric Psychology, 36, 1030–1042. 10.1093/jpepsy/jsr032 21693542

[bjhp12510-bib-0059] UNAIDS Data . (2019). Retrieved from https://www.unaids.org/sites/default/files/media_asset/2019‐UNAIDS‐data_en.pdf

[bjhp12510-bib-0060] Verbeek, M. (2017). Using linear regression to establish empirical relationships. IZA World of Labor. Retrieved from https://wol.iza.org/articles/using‐linear‐regression‐to‐establish‐empirical‐relationships/long

[bjhp12510-bib-0061] Wang, K., Chen, W. T., Zhang, L., Bao, M., Zhao, H., & Lu, H. (2016). Facilitators of and barriers to HIV self‐management: Perspectives of HIV‐positive women in China. Applied Nursing Research, 32, 91–97. 10.1016/j.apnr.2016.06.004 27969059 PMC5158022

[bjhp12510-bib-0062] Wisdom, J., & Creswell, J. W. (2013). Mixed methods: Integrating quantitative and qualitative data collection and analysis while studying patient‐centered medical home models. Rockville, MD: Agency for Healthcare Research and Quality. February 2013. AHRQ Publication No. 13–0028‐EF. pcmh.ahrq.gov.

[bjhp12510-bib-0063] Zhou, E., Qiao, Z., Cheng, Y., Zhou, J., Wang, W., Zhao, M., … Wang, R. (2019). Factors associated with depression among HIV/AIDS children in China. International Journal of Mental Health Systems, 13(1), 10. 10.1186/s13033-019-0263-1 30828360 PMC6381654

